# A dimer between monomers and hexamers—Oligomeric variations in glucosamine-6-phosphate deaminase family

**DOI:** 10.1371/journal.pone.0271654

**Published:** 2023-01-04

**Authors:** Sathya Srinivasachari, Vikas R. Tiwari, Tripti Kharbanda, Ramanathan Sowdamini, Ramaswamy Subramanian

**Affiliations:** 1 Institute for Stem Cell Science and Regenerative Medicine, Bengaluru, Karnataka, India; 2 National Center for Biological Sciences—Tata Institute of Fundamental Research, Bengaluru, Karnataka, India; 3 Department of Biological Sciences and Weldon School of Biomedical Engineering, Purdue University, West Lafayette, Indiana, United States of America; Ahmadu Bello University, JAPAN

## Abstract

In bacteria that live in hosts whose terminal sugar is a sialic acid, Glucosamine-6-phosphate deaminase (NagB) catalyzes the last step in converting sialic acid into Fructose-6-phosphate. These bacteria then use the Fructose-6-phosphate as an energy source. The enzyme NagB exists as a hexamer in Gram-negative bacteria and is allosterically regulated. In Gram-positive bacteria, it exists as a monomer and lacks allosteric regulation. Our identification of a dimeric Gram-negative bacterial NagB motivated us to characterize the structural basis of two closely related oligomeric forms. We report here the crystal structures of NagB from two Gram-negative pathogens, *Haemophilus influenzae (Hi)* and *Pasturella multocida (Pm)*. The *Hi*-NagB is active as a hexamer, while *Pm*-NagB is active as a dimer. Both Hi-NagB and Pm-NagB contain the C-terminal helix implicated as essential for hexamer formation. The hexamer is described as a dimer of trimers. In the Pm-NagB dimer, the dimeric interface is conserved. The conservation of the dimer interface suggests that the three possible oligomeric forms of NagB are a monomer, a dimer, and a trimer of dimers. Computational modeling and MD simulations indicate that the residues at the trimeric interface have less stabilizing energy of oligomer formation than those in the dimer interface. We propose that Pm-NagB is the evolutionary link between the monomer and the hexamer forms.

## 1 Introduction

Sialic acids are nine-carbon acidic sugars universally present on all mammalian cell surfaces as terminal sugars of glycoproteins and glycolipids [1]. Bacteria colonizing the heavily sialylated mammalian gut and respiratory tract have evolved unique mechanisms for scavenging the sialic acid from the host and using it as an energy source for survival. Apart from this, commensal pathogens like *H*. *influenza*, *F*. *nucleatum*, *P*. *multocida*, and other opportunistic pathogens have also found ways to sugar-coat their cell surfaces with sialic acid and use it for molecular mimicry, thereby evading human defense mechanisms [2–4]. Several reports show that the complex regulatory interplay of the sialometabolic genes significantly influences the colonization and pathogenicity of commensal pathogens. Sialyation is also a major virulence determinant of biofilm formation *in H*. *influenzae* and *P*. *multocida*. *H*. *influenzae* causes otitis media, and *P*. *multocida* is zoonotic and infects several animals and birds. Sialic acid (Neu5Ac) scavenged from the host is catabolized into Fructose-6-phosphate by a series of enzymes. The final step is the conversion of D-glucosamine-6-phosphate to D-fructose-6-phosphate (F-6-P) and ammonia. This reaction is catalyzed by the enzyme Glucosamine-6-phosphate deaminase (NagB), an aldose-ketose isomerase that is tightly regulated and is part of the conserved *nag-nan* operon of many Gram-negative bacteria [3, 5]. F-6-P then enters the glycolytic cycle as a carbon source for the bacteria to survive in the sialic acid rich niche of the host. Studies in *S*. *mutans* show that NagB inactivation decreases the expression of virulence factors and impedes biofilm formation and saliva-induced aggregation [6]. Similarly, NagB deletion mutants in *S*. *pneumoniae* and *B*. *subtilis* indicate that NagB is vital for growth using sialic acid as the sole carbon source [7, 8]. These suggest the critical role that NagB plays in pathogen survival.

**Fig 1:**
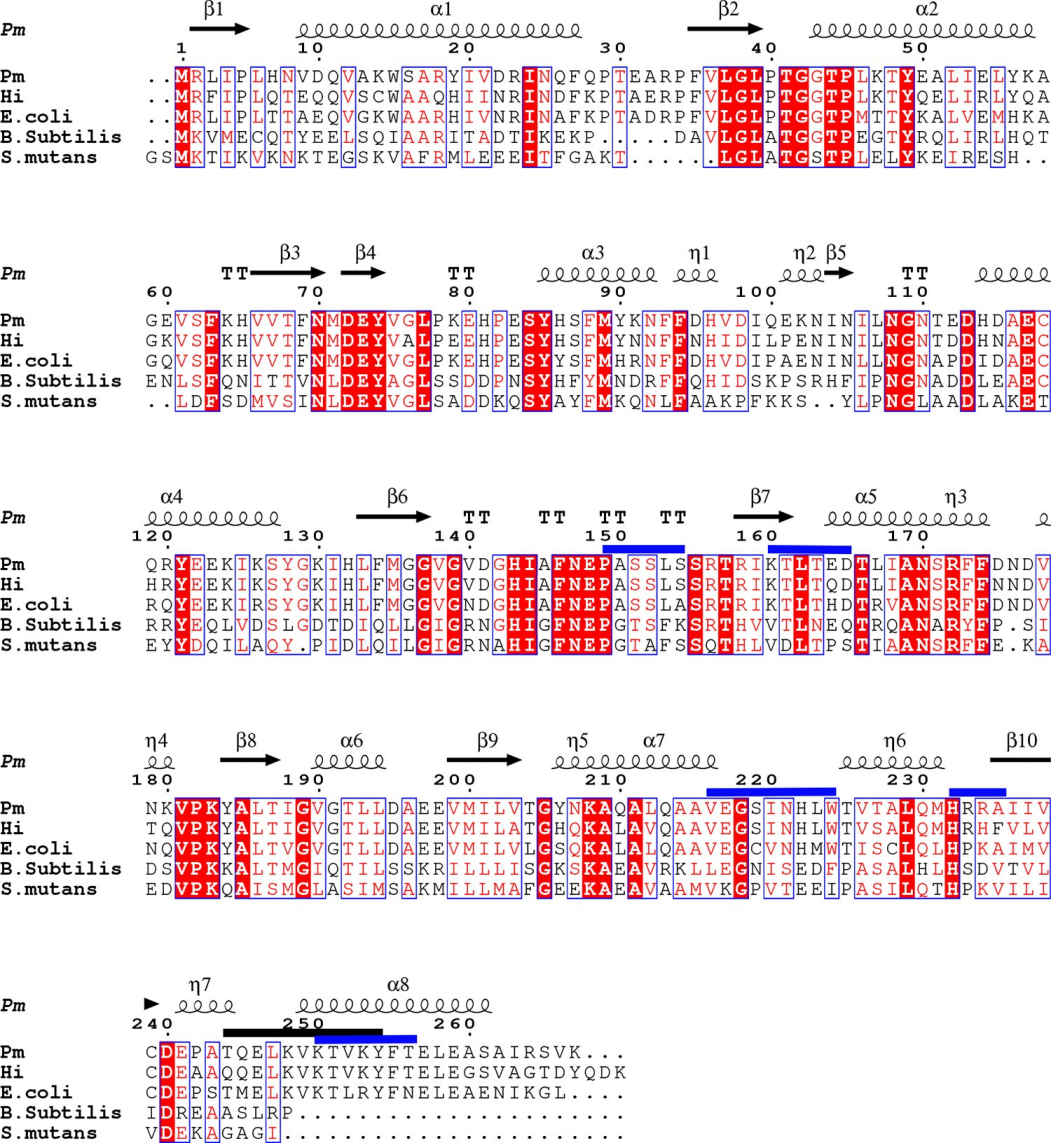
Multiple sequence alignment of NagB from *E*.*coli*, *P*.*multocida*, *H*.*influenza*, *B*.*subtilis* and *S*.*mutans* generated using ESPript 3.0. The secondary structural elements are marked based on the structure of Pm-NagB. The residues marked in black bars are residues in the dimer interface. The residues marked in blue bars are the residues in the interface of subunits in the trimer interface.

Protein structures are often oligomers, and the oligomeric state of the protein has implications for its function. Usually, proteins with similar sequences that catalyze the same reaction have similar quaternary structures. In the sialic acid catabolism pathway, we recently reported on the variations in the quaternary structure of *N*‐acetylglucosamine-6‐phosphate deacetylase (NagA) between two closely related sequences, one from *E*. *coli* and the other from *Pasteurella multocida* [9]. Structures of NagB from Gram-positive bacteria, *E*.*coli*, and human enzymes have been reported [7, 10–12]. The structures fall into two classes hexameric (dimer of trimers as described by earlier authors) enzymes that are allosterically regulated and monomeric enzymes. N-acetylglucosamine-6-phosphate (GlcNAc6P) is the allosteric regulator of the hexameric enzymes [10, 13]. The monomeric form found in Gram-positive bacteria does not show allostery [7, 12]. When the first structure of the monomeric form was determined, sequence comparisons between the different quaternary structural variations of NagB were carried out (Fig 3 of reference 7). A C-terminal helix was a common feature of all the hexameric variants. The monomeric variants did not have this C-terminal helix. That conclusion would suggest that NagB from *P*. *multocida* and *H*. *influenzae* would be hexameric. However, given the quaternary structural variation of NagA, the enzyme that catalyzes the conversion of *N*‐acetylglucosamine-6‐phosphate to glucosamine-6‐phosphate (the substrate of NagB), we hypothesized that the next enzyme in the pathway NagB would also show interesting variations in quaternary structure. This paper focuses on investigating the structure of the deaminases from *H*. *influenza* (*Hi*) and *P*. *multocida* (*Pm*) (both Gram-negative bacteria) and the comparison of these structures with other known NagB structures. Our studies show that HiNagB is a hexamer. Despite very high sequence similarity, conserved active site, and the presence of the C-terminal helix, Pm NagB is a dimer (Fig 1).

## 2 Materials and methods

### 2.1 Protein expression and purification (*Hi*NagB and *Pm*NagB)

Proteins were expressed from the recombinant plasmids, synthesized using gateway cloning technology [14], in *Escherichia coli* BL21(DE3)* cells. The cells were grown in Luria Bertani medium containing ampicillin (100ug/mL) at 37°C to an OD_600_ of 0.6. The cells were induced with 0.3 mM IPTG. After induction, the cells were allowed to grow for three hours at 37°C. The cells were harvested and centrifuged at 6000 rev min ^-1^ for 30 mins at 4°C. Each 1 L cell pellet was resuspended in 25 mL of resuspension buffer (20 mM HEPES, 150 mM NaCl, 5 mM Imidazole, pH 8.0) with protease inhibitor cocktail without EDTA (Roche). The cells were lysed using an emulsiflex at 100 MPa. The lysate was centrifuged at 13000 rev min^-1^ for 45 mins. The supernatant was purified using Ni-NTA affinity chromatography. A buffer solution containing 20 mM HEPES buffer, pH 8.0, 150 mM NaCl, and 300 mM Imidazole was used to elute *Hi*NagB and *Pm*NagB from the column. The proteins were purified using size exclusion chromatography using an S200 Superdex (GE HiLoad 16/600) column with a buffer solution of 20 mM Sodium phosphate, pH 7.4, 50 mM NaCl, and 10% glycerol. Purified proteins were concentrated to 15–16 mg/mL for crystallization. Size exclusion chromatography was also carried out in 50 mM HEPES buffer, with 150 mM NaCl, 5mM beta-mercaptoethanol, pH 8.0. From 2 L of E. coli, we obtained about 31 mg and 15 mg of crystallization quality protein of *Hi*NagB and *Pm*NagB, respectively.

### 2.2 Crystallization, data collection, and processing

Hanging-drop vapor-diffusion experiments were performed using a Mosquito robot (TTP Labtech). Crystals of *Pm*NagB and *Hi*NagB were obtained by mixing 0.5 μl screening solution with 0.5 μl of protein solution (15–16 mg/mL) and equilibrating the mixture against 100 μl of commercially available crystallization screen conditions (Crystal Screen, Hampton Research). Rod-shaped crystals appeared for *Pm*NagB within 15 days at 18°C in the presence of 100 mM Sodium Cacodylate/HCl pH 6.5, 200 mM MgCl_2_, and 20% PEG 1000. Cuboid-shaped crystals appeared for *Hi*NagB within 15 days at 18°C in the presence of 2% w/v.

Tasciminate pH 5.0, 0.1 M Sodium Citrate tribasic pH 5.6, 16% PEG 3350 with 0.1 M Strontium Chloride. 6H_2_O as additive.

Diffraction data were collected to 2.3 Å resolution from a single crystal of *Pm*NagB and 3.0 Å resolution from a single crystal of *Hi*NagB on the PROXIMA-1 beamline at the SOLEIL synchrotron source. Data were processed with XDS/XSCALE [15] and scaled with AIMLESS from the CCP4 suite [16]. The data-processing statistics are provided in Table 1.

**Table 1:** Data-processing and refinement statistics. Values in parentheses are for the highest resolution shell.

Data Set	HiNagB	PmNagB
Data processing		
Space group	P2_1_	P2_1_
*a*, *b*, *c (Å)*	103.41 144.30 131.14	84.80 79.57 85.30
*⍺*, *ß*, *Ÿ (°)*	90.00 92.07 90.00	90.00 109.13 90.00
Wavelength (Å)	0.98	0.98
Resolution (Å)	48.67–3.0 (3.1)	41.91–2.30 (2.4)
*R*_merge_	0.170(0.530)	0.036 (0.203)
*R*_p.i.m_.	0.11 (0.34)	0.02 (0.14)
Completeness (%)	99.5 (99.4)	99.2 (99.35)
Mean I/sigma(I)	6.45 (2.42)	17.96 (4.41)
CC1/2	0.98 (0.79)	0.99 (0.98)
Total No. of reflections	143943 (14006)	90060 (8289)
No. of unique reflections	76608	47456
Multiplicity	1.9 (1.8)	1.9 (1.8)
*B* factor from Wilson plot (Å^2^)	46.1	42.7
Refinement statistics		
Resolution (Å)	38.78–3.0 (3.1–3.0)	37.1–2.3 (2.4–2.3)
No. of reflections	76519	47397
No. of reflections, test set	3814	2275
*R*_work_*/R*_free_ (%)	0.19/0.24	0.20/0.23
No. of non-H atoms		
Protein	3132	1072
macromolecules	25020	8517
R.m.s. deviations		
Bond lengths (Å)	0.008	0.004
Bond angles (°)	1.30	1.01
Average **B** factors (Å^2^)		
Overall	45.56	51.30
Ramachandran plot		
Favored (%)		
Outliers (%)	97	96
PDB-ID	0	0
	7LQN	7LQM

### 2.3 Structure solution and refinement

The phases of the *Hi*NagB and *Pm*NagB structures were obtained by molecular replacement using Phaser-MR in the PHENIX suite [17, 18]. The search models were monomeric polyalanine models of PDB entry *1dea* (Gluocosamine-6-phosphate-deaminase from *E*. *coli*). Model building was carried out in Coot [19], and the structures were iteratively refined using the PHENIX Suite. Water molecules were automatically added during the refinement process but were manually checked for hydrogen bonding and density fit to both (2|Fo|- |Fc|) and (|Fo| -|Fc|) maps. The structure-solution and refinement statistics are provided in Table 1.

### 2.4 Electron cryomicroscopy

Protein was purified as described for crystallography. 3 μl of *Hi*NagB at 1 mg/ml were applied to Quantifoil holey carbon grids (R 1.2/1.3, Au 300 mesh) with blotting and freezing accomplished on a Vitrobot mark IV at 18°C and 100% RH. The images of these grids showed that NagB particles are well separated with high contrast. The data of NagB were collected on Titan Krios with a Falcon 3 detector in counting mode at 1.07 Å/pixel sampling with images exposed for 60 seconds with a total accumulated dose of ~30 e^-^/Å^2^ and dose fractionated into 25 frames, with each frame having a dose of ~1.2 e^-^/Å^2^. An algorithm within Relion3 was used for full-frame alignment and dose weighting [20]. The motion-corrected images were imported into CryoSPARC [21]. After picking spots manually from 9 frames followed by 2D classification, we selected 2D templates that seemed good. Then template picking was carried out on the 470 images. The picked spots were manually curated, resulting in a total of 54243 particles. 2D classification, followed by ab initio reconstruction and homogeneous refinement, resulted in maps with an overall resolution of 6.2 Å with no symmetry and 4.3 Å with the application of D3 symmetry.

### 2.5 MD simulation and analysis

We used Molecular Dynamics (MD) simulations to computationally test our hypothesis and elucidate the roles of residues in the oligomerization of NagB. The *Pm*NagB-hexamer and *Hi*NagB-hexamer structures were subjected to molecular dynamics simulation for 50 nanoseconds using the Desmond package of Schrodinger [22–24]. Protein structures were prepared using the protein preparation wizard of Maestro (Maestro, Schrödinger, LLC, New York, NY, 2019). In System Builder, the TIP4P model was specified for water molecules, and an orthorhombic box shape was used with a buffer distance of 10 Å, followed by a minimization of the box size. The system was neutralized, and salt (NaCl) was added. The solvated system was subjected to the default relaxation protocol of Desmond before the production MD run. The relaxation protocol involves energy minimization steps using the steepest descent method with a maximum of 2000 steps. The energy minimization is done with the solute being restrained using 50 kcal/mol/Å force constant on all solute atoms and without restraints. Energy minimization is followed by short MD simulation steps which involve: 1) simulation for 12 picoseconds at 10K in NVT ensemble using Berendsen thermostat with restrained non-hydrogen solute atoms, 2) simulation for 12 picoseconds at 10K and one atmospheric pressure in NPT ensemble using Berendsen thermostat and Berendsen barostat with restrained non-hydrogen solute atoms, 3) simulation for 24 picoseconds at 300K and one atmospheric pressure in NPT ensemble using Berendsen thermostat and Berendsen barostat with restrained non-hydrogen solute atoms, and 4) simulation for 24 picoseconds at 300K and one atmospheric pressure in NPT ensemble using Berendsen thermostat and Berendsen barostat without restraints. After relaxation, production MD was run in NPT ensemble using OPLS 2003 force field [25]. For simulations, default parameters of RESPA integrator (2 femtoseconds time step for bonded and near non-bonded interactions while six femtoseconds for far non-bonded interactions) were used. The temperature and pressure were kept at 300K and one bar using the Nose-Hoover chain method and the Martyna-Tobias-Klein method [26], respectively. The production MD was run for 50 nanoseconds. MD simulation analysis was done using the Desmond module’s simulation interaction diagram (SID) and Simulation event analysis (SEA) packages.

## 3 Results and discussion

### 3.1 Structure of *Hi*NagB

*Hi*NagB crystallized in the P 2_1_ space group with 12 monomers in the asymmetric unit. The Matthews coefficient is 2.35 with 47.6% solvent content. The structure of *Hi*NagB was determined to 3.0 Å resolution with the final refined R-factor and R-free of 0.194 and 0.242, respectively (Table 1). The PDB ID assigned for PmNagB is 7LQN.

### 3.2 Structure of *Pm*NagB

*Pm*NagB crystallized in the P 2_1_ space group with 4 monomers in the asymmetric unit, Matthews coefficient is 2.16 with 42.6% solvent content. The structure of *Pm*NagB was determined to 2.3 Å resolution with the final refined R-factor and R-free of 0.20 and 0.23, respectively (Table 1). The PDB ID assigned for *Pm*NagB is 7LQM. Analysis of the crystal packing reveals that the protein is a dimer. The dimeric form also corresponds to results obtained from Size Exclusion Chromatography experiments carried out in the buffer used for crystallization (Sodium Phosphate buffer containing glycerol) and in HEPES buffer with no glycerol.

### 3.3 Overall fold and comparison with other proteins

**Fig 2:**
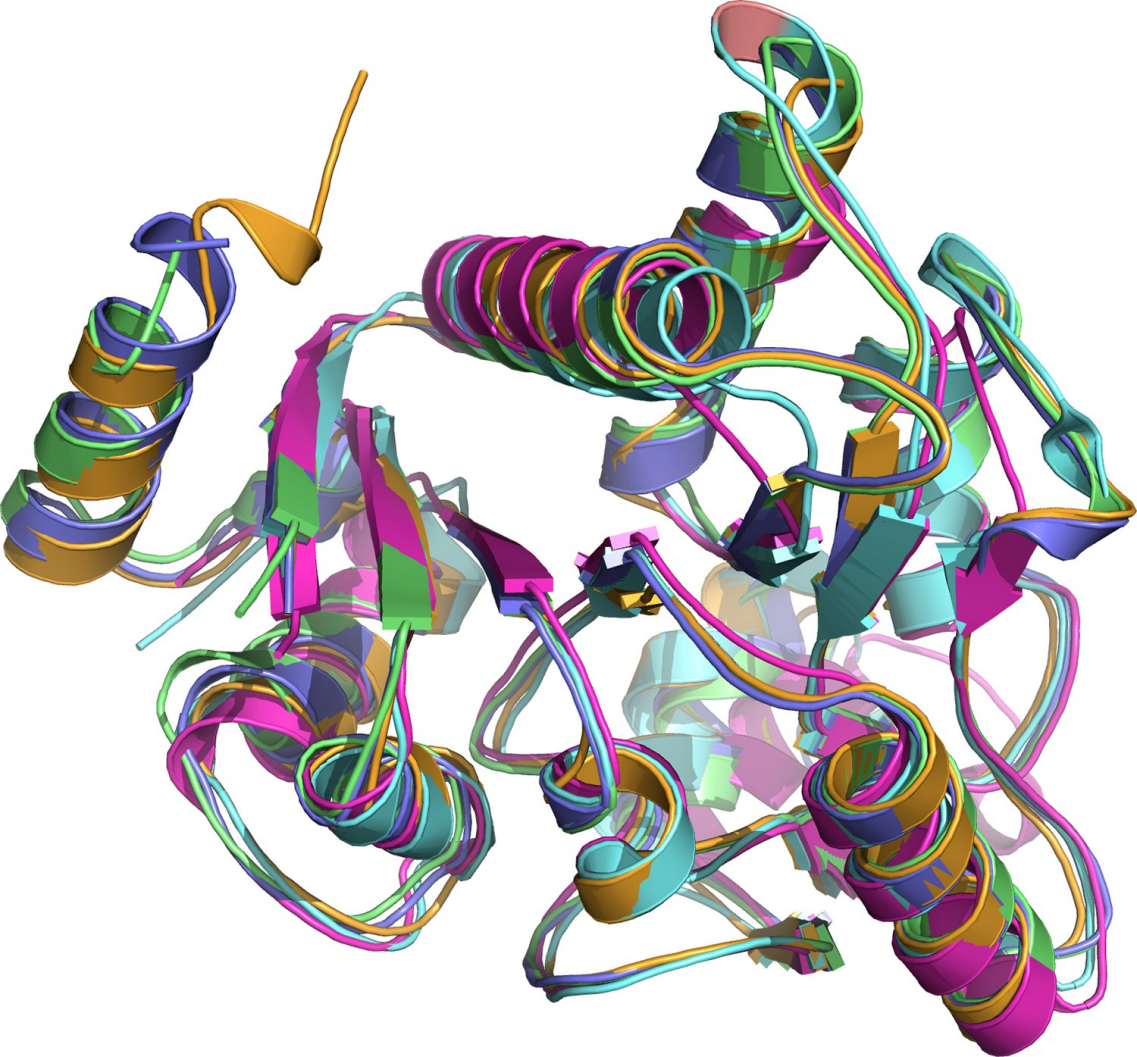
Overlay of deaminase monomers: Magenta- *B*.*subtilis*, cyan- *S*.*mutans*, orange- *E*.*coli*, violet- *Hi and* green: *Pm*. The presence of alpha-8 helix (in *Pm*, *Hi* and *E*. *coli*) is seen on the top left.

The monomer folds of both *Hi*NagB and *Pm*NagB are conserved and resemble the *E*.*coli*NagB open structure with seven-stranded parallel β sheets surrounded by eight α helices (Fig 2). The alpha-8 helix is also conserved in *Hi* and *Pm*, which is seen missing in the case of the monomeric deaminases from *B*.*subtilis* and *S*.*mutans* (Fig 2) [7, 12].

The overall topology of the monomer resembles a modified NADH-binding domain similar to *E*.*coli*NagB [13]. The allosteric site is required for GlcNAc6P to bind and allosterically regulate the enzyme [10]. The overall structure of the monomers, when superposed, suggests that the root mean square deviation between them is low, and the largest deviation observed is about 1.0Å for all the C-alpha atoms. Note that the two monomeric forms of the protein (from *Streptococcus mutans* and *Bacillus subtilis*) lack the C-terminal helix (Fig 2). Table 2 lists the RMS deviations between the different proteins.

**Table 2:** Superposition of the C-alpha atoms of monomers of the different bacterial NagBs whose structures have been determined. The RMS deviations are in Å units. The numbers in the parenthesis are the number of C-alpha atoms that were superposed. Superposition was carried out using the ‘super’ command in Pymol. The two monomeric proteins have a C-terminal helix missing. Hex- in the names corresponds to proteins that are hexamers, and mono- for those that are monomeric.

	H.flu (hex)	Bb (hex)	E.coli (hex)	Pm(dimer)	Sm (mono)
H.flu (hex)					
Bb (hex)	0.50(210)				
E.Coli (hex)	0.48(228)	0.57(207)			
Pm (dimer)	0.41(218)	0.58(230)	0.44(232)		
Sm (mono)	0.87(176)	0.69(177)	1.0(190)	0.86(181)	
Bs (mono)	0.99(220)	1.0(218)	1.0(218)	0.94(218)	0.97(185)

H.flu–*Haemophilus influenzae* (7lqn); Bb—*Borrelia burgdorferi*. (3hn6); E. coli–*Escherichia coli*. (1fsf); Pm–*Pasteurella multocida* (7lqn); Sm–*Streptococcus mutans* (2ri0); Bs–*Bacillus subtilis* (2bkv).

### 3.4 Quarternary variations—hexameric and dimeric deaminases

**Fig 3:**
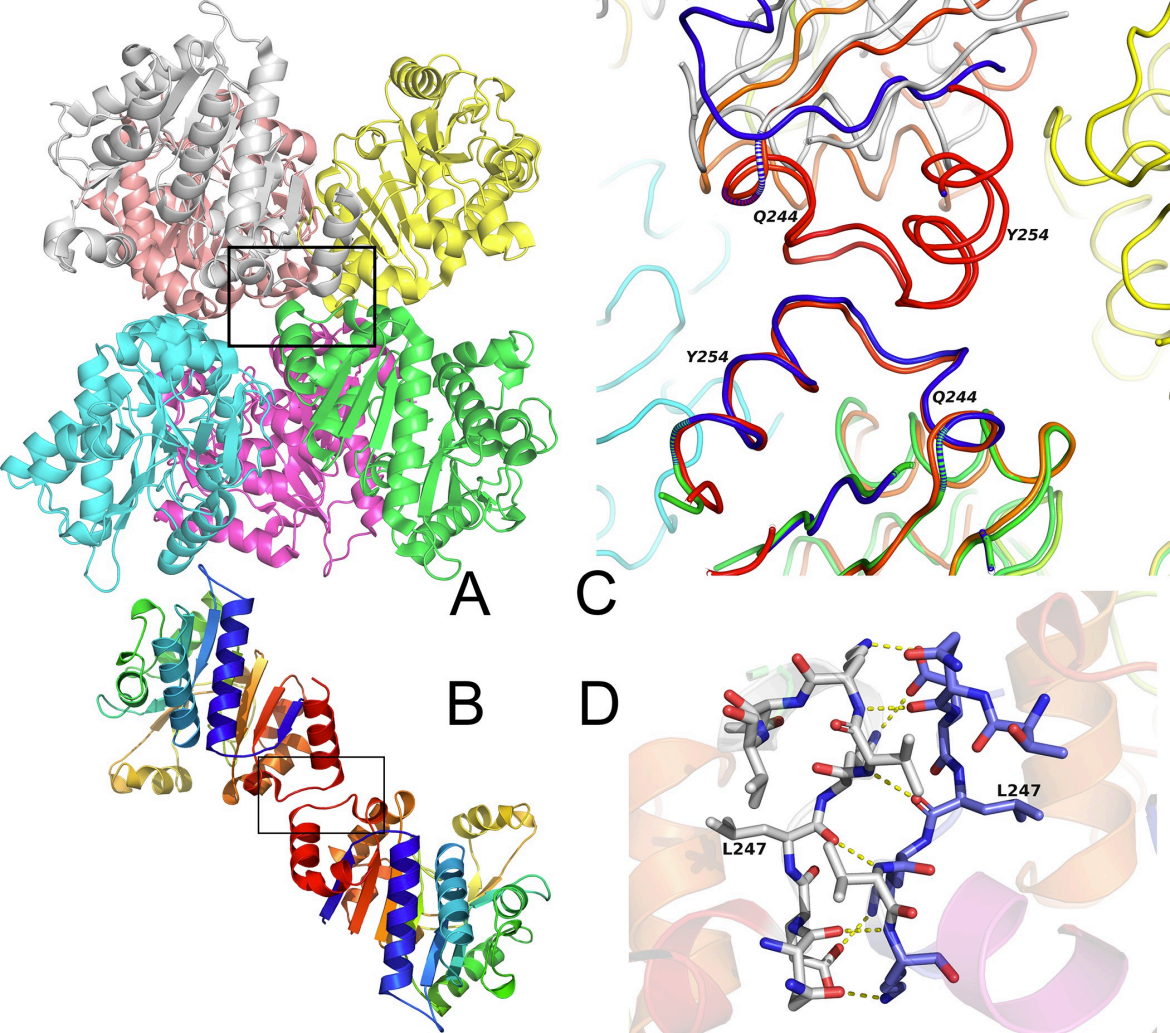
Overall structure. A: Cartoon representation of the *Hi*NagB hexamer. Each monomer is colored differently. The protein is a trimer of dimers and the orientation shows three subunits at the bottom and three at the top. The black box is the dimer interface. B: Cartoon representation of the *Pm*NagB dimer. Each monomer is colored as a rainbow–with the N-terminus in Blue and the C-terminus in Red. The black box is the dimer interface. C: The zoomed-up area of the dimer interface–the area that is approximately marked in the black box. One of the subunits of *Pm*NagB is superposed on one of the subunits of *Hi*NagB (the green subunit in 3A). Notice that the overlapped subunits superpose well, but the other monomer of the dimer is shifted. D: The interaction of the residues in the dimer interface. There are several main chain hydrogen bonds.

*Hi*NagB forms a hexameric structure (trimer of dimers) (Fig 3A) similar to *E*.*coli*NagB. The interactions between two trimers to form a hexamer are similar to that observed in *E*.*coli*NagB [11]. The residues in the trimeric interface are also conserved. It is important to note that even though *Pm*NagB has 80% sequence identity with *Hi*NagB and *E*.*coli* NagB, with all the active site residues and most of the interface residues conserved among the three deaminases, *Pm*NagB forms a dimer and not a hexamer (Fig 3B). Fig 3C shows the superposition of the *Pm*NagB dimer on the *Hi*NagB hexamer and the conserved dimer interface.

To ensure that the purified proteins with quaternary structures were active deaminases, we conducted steady-state kinetic studies to check the activity of the enzymes. We used an ammonia Assay Kit–Modified Berthelot, Colorimetric detection from Abcam (ab102509), to study the release of ammonia. The results showed that the enzymes were active. A comparison of the K_M_ values of the deaminases revealed very similar values (Table 3). We calculated apparent kcat values—around 2 s^-1^ for HiNagB and 1 s^-1^ for PmNagB. A complete enzyme kinetic characterization is beyond the scope of this manuscript. However, these studies were carried out to show that the structural work on the enzymes reported here is an active enzyme.

**Table 3:** Comparison of quaternary structure and the K_M_ of different deaminases.

	Quaternary Structure	K_M_ (mM)
*E*.*coliNagB (R-state)*	Hexamer	0.55 ± 0.03 [27]
*Pm*NagB	Dimer	0.96 ± 0.2
*Hi*NagB	Hexamer	0.83 ± 0.3
*B*.*subtilis*	Monomer	0.13 ± 0.02 [8]
*S*.*mutans*	Monomer	0.21 ± 0.03 [25]

### 3.5 Crystal vs cryo-EM–A comparative study

**Fig 4:**
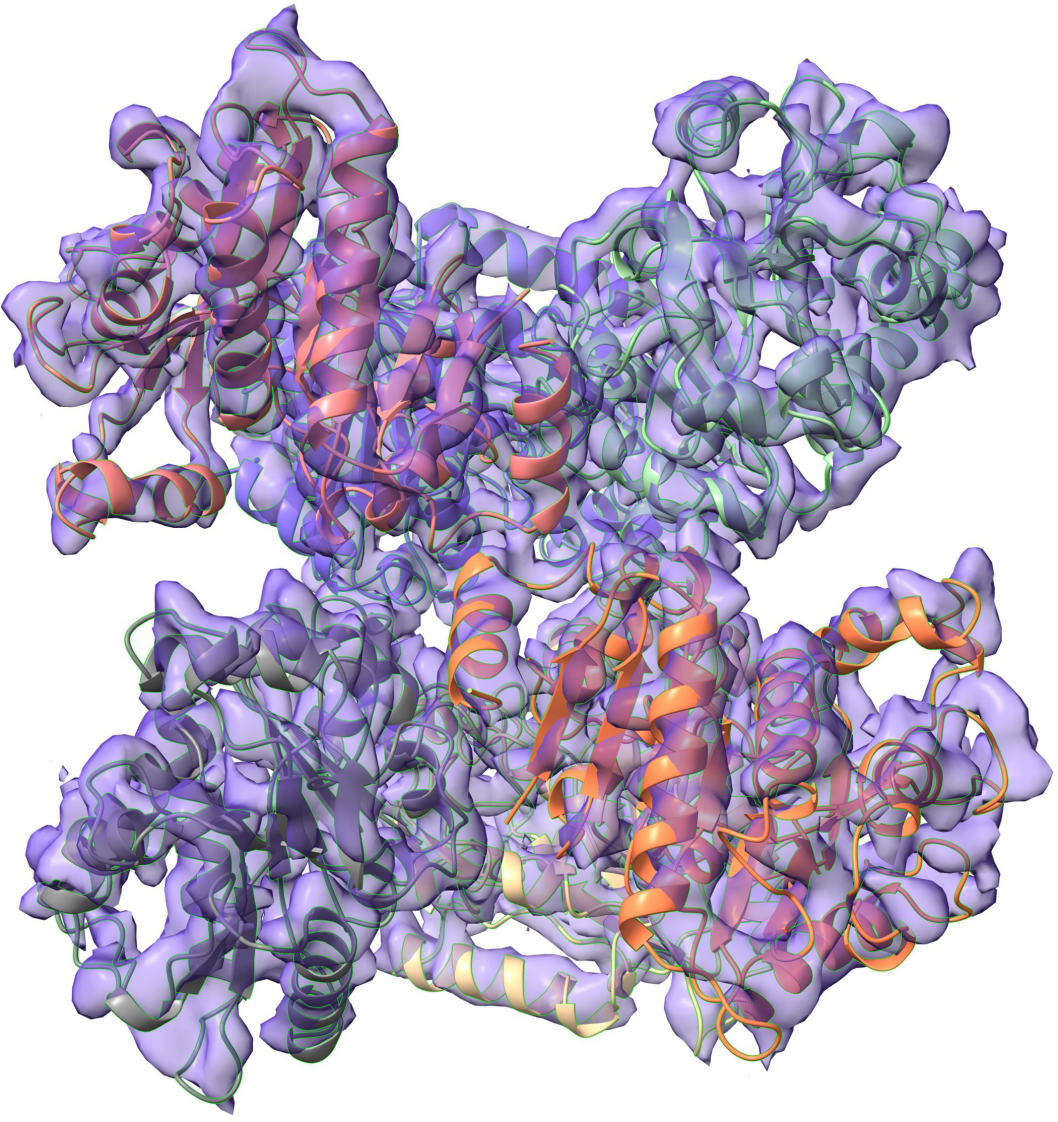
The reconstructed cryo-EM map. The crystallographic hexamer model was fit into the map. The model fits the map well and confirms the hexameric nature of HiNagB.

We further investigated whether the hexamer seen in the *Hi*NagB crystal structure is also preserved in the solution. We made cryoEM grids and collected data. Using this data, we calculated 2D class averages, and performed a 3D reconstruction. A significant number of the particles were in preferred orientations in the images. This resulted in a lower than expected resolution of the final reconstructed maps. It was clear from the initial map that the molecule was a hexamer or a trimer of dimers as anticipated. Homogeneous refinement with D3 symmetry improved the resolution from 6.2 to 4.3 Å. We then used Chimera to fit the hexamer model from crystal structure into the reconstructed 4.3 Å resolution map (Fig 4) [28]. The cryo-EM data analysis confirmed that the hexameric architecture of *Hi*NagB is conserved both in the crystal environment and in the solution.

### 3.6 Bioinformatics analysis of the interfaces

To understand the molecular basis for the observed difference in the quaternary structure of *Hi*NagB and *Pm*NagB, we investigated the dimer and the trimer interfaces of *Hi*NagB, *Pm*NagB, and *E*.*coli*NagB using multiple computational tools. Using one of the monomers of *Pm*NagB-dimer with structural superposition to the *Hi*NagB-hexamer, we forcefully built a hexamer model of *Pm*NagB (*Pm*NagBHexa*) to understand the energetic of such forced hexamers computationally. Energy minimization was performed on this modeled *Pm*NagBHexa* with 100 steps of steepest descent in Swiss-PDB Viewer [24]. Then, we computed the interface pseudo energy for the dimer and trimer interfaces of *E*.*coli*NagB, *Hi*NagB-hexamer, and the minimized *Pm*NagBHexa* (the forcefully modeled hexamer) using PPCheck [29].

First, we analyzed the trimeric interface and observed that the interface stabilizing energy for *Hi*NagB and *E*.*coli*NagB was -100 kJ/mol and -170 kJ/mol, respectively. However, the calculated trimeric interface stabilizing energy for *Pm*NagBHexa* was only around -33 kJ/mol. Here, the values with less negative energies refer to poor stabilization, and values with higher negative energies refer to better stabilization, showing that *Pm*NagBHexa* is unstable as a hexamer. A closer look at the trimeric interface residues of *Hi*NagB and *Pm*NagBHexa*shows most of the residues were conserved, except two residues, namely Gln164 in *Hi*NagB is Glu in *Pm*NagB, Gln210 in *Hi*NagB is Leu in *Pm*NagB (Fig 1). Interestingly there is no consensus amino acid at position 164 between the monomeric, dimeric and hexameric versions. However, at position 210, the hexameric proteins have a Leu, the monomeric proteins have a Glu, and the dimeric protein (*Pm*NagB) has a Gln.

To investigate the crucial role of these two residues on trimer formation, we performed in-silico mutations of these two residues, E164Q-Q210L, in the trimeric interface of the *Pm*NagBHexa*, to those found in *Hi*NagB followed by minimization (100 sd step), and calculated the interface energy. The results suggested that the mutations did not yield any significant difference in the stabilization energies.

**Fig 5:**
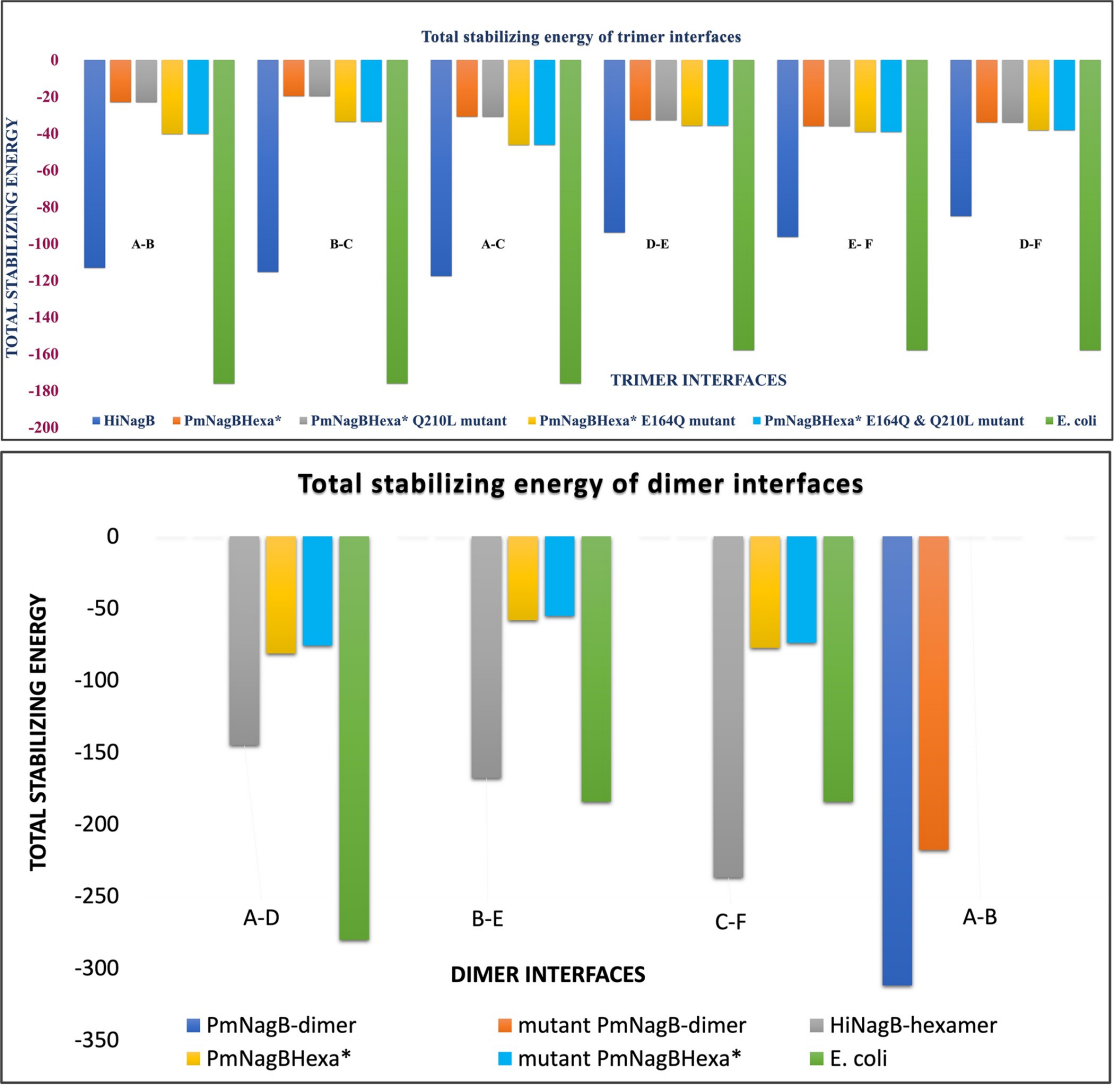
A: Total stabilizing energy of trimer interfaces of *Hi*NagB, *E*.*coli*, *PmNagB*Hexa* and its mutants (E164Q, Q210L and E164Q, Q210L double mutant) as calculated by PPCheck B: Total stabilizing energy of dimer interfaces of *Hi*NagB, *E*.*coli*, *Pm*NagBHexa* and its mutants (Y206H, P242A, T244Q triple mutant) as calculated by PPCheck.

Simultaneously, we compared the dimer and trimer interface of *Hi*NagB and *Pm*NagB (Fig 5A and 5B) to examine the contributions from the individual interface residues. The results revealed that around ten residues are not conserved between the two interfaces. The stabilization energy of *Pm*NagB as a dimer is around -300 kJ/mol, whereas that of *Hi*NagB and *Pm*NagBHexa* is around -150 kJ/mol and– 80 kJ/mol. A similar trend is noticed upon analyzing the dimer interface of *Pm*NagBHexa*, *E*.*coli*NagB, and *PmNagB*, as shown in Fig 5B. These bioinformatic data clearly show that *Pm*NagB is a dimer, not a hexamer similar to its *Hi* analog.

One would generally think a molecule made of the same sequence would crystallize in a space group where the molecular symmetry coincides with the crystallographic symmetry. In the case of *Hi*NagB, the molecule is a trimer of dimers. Interestingly, the trimer is not symmetric. The angle between subunits A and B—is 120.6°, 116.9° between A and C, and 122.5° between B and C. This results in a complete 360° rotation.

Furthermore, the relationship is not just a pure rotation from A to B but also a translation of 0.38Å along the rotation axis. In the case of A to C, the translation vector is 0.14Å, and in the case of B to C, it is 0.54Å. These are not just rotations but also include a translation along the axis. The combination of rotation and translation results in a slight asymmetry in the interactions between the A, B, and C subunits. This asymmetry is not conserved among the four different trimers in the asymmetric unit. One could say that while the protein’s oligomeric state is hexameric, there is some shear between the subunits. It is currently difficult to say if this shear impacts the activity or the function of the enzyme. The calculations of the angles and axis were performed using the draw_rotation_axis script in Pymol [30].

In the case of *Pm*NagB, the molecule is a dimer. Even though the trimer interfaces are conserved, we do not observe the interaction seen in the case of *Hi*NagB and *E*.*coli*NagB. Fig 3D shows the interface interactions that favor dimerization. This interface plays a crucial role in the quaternary state of the protein. A number of the interactions are between main chain atoms, suggesting the possibility of variations in the side chains at this interface. The dimer interface residues might serve as a signature motif between the monomeric, dimeric, and hexameric family of deaminases that can help predict the quaternary structure of other deaminases. Quaternary variations are often either related to allostery or stability. The presence of allostery in some of the NagB enzymes would suggest that is probably the role. An important question yet to be answered is the role of these quaternary variations or allostery in the physiological function of these proteins.
